# Cellular view of metabolism: metabolic biomolecular condensates

**DOI:** 10.1111/nph.70474

**Published:** 2025-08-22

**Authors:** Evan V. Saldivar, Sterling Field, Seung Y. Rhee

**Affiliations:** ^1^ Department of Biology Stanford University Stanford CA 94305 USA; ^2^ Plant Resilience Institute Michigan State University East Lansing MI 48824 USA; ^3^ Department of Biochemistry and Molecular Biology Michigan State University East Lansing MI 48824 USA; ^4^ Department of Plant, Soil, and Microbial Sciences Michigan State University East Lansing MI 48824 USA; ^5^ Department of Plant Biology Michigan State University East Lansing MI 48824 USA

**Keywords:** bacterial microcompartment, biomolecular condensate, membraneless organelle, metabolic biocondensate, metabolon, plant cell biology

## Abstract

Recent studies across biological systems highlight an important function of biomolecular condensates (hereafter biocondensates) in regulating physiology. Biocondensates are membraneless organelles that compartmentalize cellular processes and allow regulatory control of key biomolecules through their assembly and disassembly. Biocondensates have been identified in molecular pathways ranging from RNA regulation to metabolism to seed germination in plants. In this review, we focus on biocondensates that control metabolism. Most of this review addresses metabolic biocondensates in bacteria, algae, and animals whose functions are involved in core metabolism relevant to plants, even if their existence as metabolic biocondensates has not yet been described in plants. We hope this review provides useful information for a broad audience and encourages new directions into previously characterized enzymes and pathways to understand how their subcellular localization could impact function.

**Table:** 

	Contents	
	Summary	555
I.	Introduction: Metabolic biocondensates and metabolons	555
II.	Classical examples of metabolic biocondensates	556
III.	Recently described metabolic biocondensates	558
IV.	Emerging technologies for discovering metabolic biocondensates	559
V.	Considerations for engineering metabolic biocondensates	560
VI.	Conclusion	560
	Acknowledgements	560
	References	561

## I. Introduction: metabolic biocondensates and metabolons

Biocondensates are membraneless macromolecular structures that concentrate biomolecules *in vivo* (Banani *et al*., 2017) into a physical state (solid, liquid droplet, or gel‐like) different from dispersed localization (Field *et al*., 2023). In plants, biocondensates vary in function from driving primary metabolism to regulating abiotic stress responses and development (Field *et al*., 2023). Some biocondensates function as metabolons, transiently forming complexes bringing pathway enzymes in close proximity to facilitate metabolite channeling between enzymes (Zhang & Fernie, 2020). Metabolons increase pathway efficiency by allowing metabolites to transfer from the active site of one enzyme to the active site of another enzyme without being released into solution (Zhang & Fernie, 2020). In this review, we focus on biocondensates with this function, which we call metabolic biocondensates.

Historically, metabolons and metabolic biocondensates have been discussed separately. However, the distinction between metabolons (defined by enzymatic cooperation and biochemical properties) and biocondensates (defined by biophysical properties) is more blurred than previously considered. The identification of a metabolon, including examples characterized in plants (Laursen *et al*., 2016; Mucha *et al*., 2019; Nakayama *et al*., 2019), requires first elucidating the enzymes in a pathway and then demonstrating meaningful physical interactions between pathway enzymes (Bassard & Halkier, 2018). Alternatively, for metabolic biocondensates, subcellular compartments have been observed first, then shown to have a metabolic function, and later demonstrated to act as metabolons. Examples of metabolic biocondensates are described in the next section.

## II. Classical examples of metabolic biocondensates

Initially, the discovery of metabolic biocondensates followed observation of organelle‐scale structures using microscopy (Table 1). Several of the first discovered biocondensates were bacterial microcompartments, which are conspicuous structures under transmission electron microscopy. Bacterial microcompartments are large (40–600 nm; Kerfeld *et al*., 2018) icosahedral proteinaceous compartments that physically separate pathway enzymes and intermediates from the cytoplasm. Here, the outer protein layer of a microcompartment is termed the shell. Bacterial microcompartments house diverse metabolic pathways, including catabolism of ethanol, ethanolamine, and sugar phosphates (Sutter *et al*., 2021). In the interest of space, we limit discussion of bacterial microcompartments to two well‐studied examples: the propanediol utilization (pdu) microcompartment and the carboxysome. The functional diversity of bacterial microcompartments is detailed in Sutter *et al*. (2021). Beyond bacterial microcompartments, we discuss the pyrenoid, a metabolic biocondensate discovered using light microscopy in algae.

**Table 1:** Evidence underpinning the discovery of each biocondensate.

Metabolic biocondensate	Evidence for structure or function of biocondensate	References for each stage of discovery
Pdu microcompartment	The pdu microcompartment was observed initially as an electron‐dense structure in wild‐type bacterial cells.	Shively *et al*. (1998)
	The genome locus encoding the microcompartment was later found to contain enzymes involved in 1,2‐propanediol metabolism.	Bobik *et al*. (1999)
Carboxysome	The carboxysome was observed initially as an electron‐dense structure in wild‐type bacterial cells.	Shively *et al*. (1970)
	The carboxysome was later purified and shown to contain active RuBisCO.	Shively *et al*. (1973)
Pyrenoid	The pyrenoid was initially observed using light microscopy in wild‐type algal cells.	Vaucher (1803)
	Purified pyrenoids were shown to have RuBP carboxylase activity *in vitro*.	Holdsworth (1971)
Purinosome	The purinosome was initially hypothesized following copurification of purine salvage enzymes.	Smith *et al*. (1980)
	Later, cytoplasmic speckles were confirmed for some purine salvage enzymes.	An *et al*. (2008)
	Substrate channeling was confirmed using mass spectrometry imaging.	Pareek *et al*. (2020)
G‐bodies	G‐bodies were initially discovered by tagging the proteins phosphofructokinase and enolase, which were found to form cytoplasmic aggregates *in vivo*.	Miura *et al*. (2013); Jang *et al*. (2016)
	The G‐body proteome was uncovered using Co‐immunoprecipitation Mass Spectrometry, with phosphofructokinase and enolase as bait proteins.	Miura *et al*. (2013); Jang *et al*. (2016)
Rhamnosome	*Arabidopsis thaliana* RHM1 was shown to localize to cytoplasmic speckles.	Wang *et al*. (2009)
	Formation of rhamnosome was shown to correlate with UDP‐rhamnose synthesis based on molecular genetic approaches.	Field *et al*. (2024)

pdu, propanediol utilization; RuBP, ribulose 1,5‐bisphosphate; UDP, uridine diphosphate.

### Propanediol utilization microcompartment

Some bacteria that colonize mammals can grow on highly reduced carbon sources from the environment, including 1,2‐propanediol (Toraya *et al*., 1978; Jeter, 1990). To accomplish this, they contain a microcompartment that houses the pathways required for the conversion of 1,2‐propanediol into 1‐propanol or propionyl‐PO_4_^2−^ (Bobik *et al*., 1999). This propanediol utilization (pdu) microcompartment is best described in *Salmonella enterica*, although components of the pdu microcompartment have been identified in a wider range of bacteria (Sutter *et al*., 2021). The pdu microcompartment contains enzymes in three pathways that degrade 1,2‐propanediol and facilitates efficient utilization of 1–2 propanediol through its selective permeability (Jakobson *et al*., 2017).

**Fig. 1:**
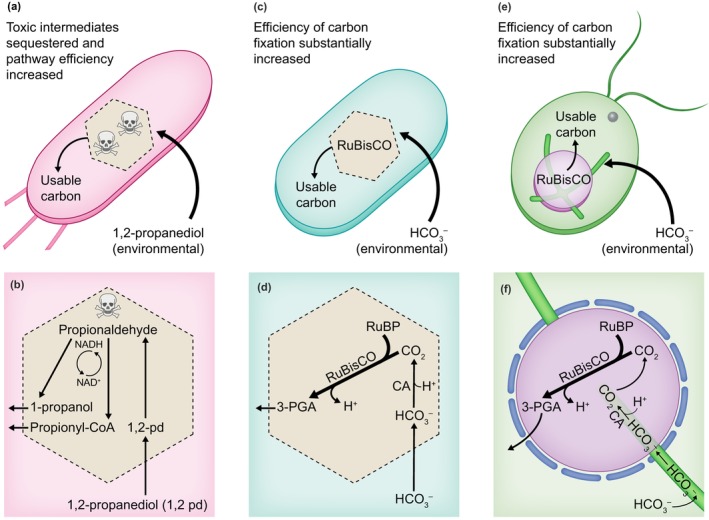
Physiological and cellular view of three classically studied metabolic biocondensates. An illustration of the function of the 1,2‐propanediol utilization (pdu) microcompartment (a, b), the carboxysome (c, d), and pyrenoid (e, f) at the whole‐cell physiological level (a, c, e) and metabolic pathway level (b, d, f). The pdu microcompartment permits the efficient use of 1,2‐propanediol without releasing the toxic intermediate, propionaldehyde, into the cytoplasm. The carboxysome permits efficient carbon fixation at low carbon dioxide (CO_2_) by converting bicarbonate (HCO_3_^−^) into CO_2_ in a biocondensate that contains ribulose‐1,5‐bisphosphate carboxylase/oxygenase (RuBisCO). It has been proposed that the carboxysome shell serves as a diffusion barrier to gaseous CO_2_ and O_2_. By rapidly converting HCO_3_^−^ into CO_2_, cells maintain a strong gradient of HCO_3_^−^ ions across the carboxysome shell, facilitating diffusion. The pyrenoid, in conjunction with the thylakoid tubules (shown in green), delivers CO_2_ to RuBisCO using a luminal carbonic anhydrase (CA). The carbonic anhydrase is active in the lumen of the thylakoid. Additional metabolite abbreviations include oxygen (O_2_), proton (H+), oxidized nicotinamide adenine dinucleotide (NAD+), reduced nicotinamide adenine dinucleotide (NADH), 3‐phosphoglycerate (3‐PGA), 2‐phosphoglycolate (2‐PG), and ribulose 1,5‐bisphosphate (RuBP). Arrows in (a), (c), and (e) represent the transport of metabolites into and out of the metabolic biocondensates. Arrows in (b), (d), and (e) represent enzymatic reactions.

The committed step of 1,2‐propanediol utilization converts 1,2‐propanediol into propionaldehyde, a toxic intermediate (Sampson & Bobik, 2008). Downstream, propionaldehyde is oxidized to form propionyl‐PO_4_^2−^ or reduced to form 1‐propanol. The oxidation of propionaldehyde reduces NAD^+^ to NADH, while reduction of propionaldehyde oxidizes NADH to NAD^+^. Thus, NADH and NAD^+^ are cycled to sustain propionaldehyde consumption in the pdu microcompartment (Fig. 1). By concentrating enzymes sufficient for metabolism of 1,2‐propanediol, the pdu microcompartment facilitates efficient metabolite channeling and avoids introducing toxic intermediates to the cytoplasm.

### Carboxysome

Another microcompartment initially found microscopically is the carboxysome, which is a specialized microcompartment found primarily in cyanobacteria and some proteobacteria (Sutter *et al*., 2021). Carboxysomes act in the carbon concentrating mechanism (CCM) to permit efficient photosynthesis. Together with active bicarbonate (HCO_3_^−^) transport across the cellular membrane, the carboxysome increases efficiency of ribulose‐1,5‐bisphosphate carboxylase/oxygenase (RuBisCO) by concentrating CO_2_ around its active site (Price & Badger, 1991). RuBisCO can catalyze the carboxylation or oxygenation of ribulose‐1,5‐bisphosphate (RuBP). The latter reaction produces 2‐phosphoglycolate, which is converted back into RuBP through an energetically costly process termed photorespiration. To limit photorespiration, carboxysomes condense RuBisCO and carbonic anhydrase in a proteinaceous shell. The carboxysome increases the relative ratio of CO_2_ to O_2_, around Rubisco's active site, favoring carbon fixation (Price & Badger, 1991). The increased local concentration of RuBisCO and carbonic anhydrase promotes synergy between the two enzymes. Recent modeling suggests that the RuBisCO reactions, when performed in a concentrated organelle, produce protons at a sufficient rate to serve as a substrate for the conversion of HCO_3_^−^ to CO_2_ by carbonic anhydrase (Long *et al*., 2021; Fig. 1). Production of protons by RuBisCO occurs within the carboxysome and is a distinct phenomenon from the production of protons by the photosynthetic electron transport.

Carboxysomes are considered the key innovation that allowed cyanobacteria to survive the global depletion of CO_2_ over geological time (Rae *et al*., 2013). Supporting this notion, genetic ablation of the carboxysome is lethal at ambient CO_2_ (Cameron *et al*., 2013). Carboxysomes have been maintained in all tested extant lineages of cyanobacteria and multiple lineages of proteobacteria, further highlighting the importance of this metabolic biocondensate (Axen *et al*., 2014; Sutter *et al*., 2021).

### Pyrenoids

The CCM in many unicellular eukaryotic algae relies on the pyrenoid – a membraneless organelle formed via liquid–liquid phase separation (Barrett *et al*., 2021). First observed in the 19^th^ century microscopically, the pyrenoid is located within the chloroplasts of algae and boosts CO_2_ levels around RuBisCO to promote efficient carbon fixation under low CO_2_ conditions (Wang *et al*., 2015). Pyrenoids, found across eukaryotic algae and diatoms, are highly diverse in structure (reviewed in He *et al*., 2023). Here, we focus our discussion on the best‐studied pyrenoid, found in *Chlamydomonas reinhardtii*. Pyrenoids in *Chlamydomonas reinhardtii* are surrounded by starch plates that are essential for the proper localization of the CCM protein, low‐CO_2_‐inducible protein B (LCIB; Toyokawa *et al*., 2020). The pyrenoid is traversed by thylakoid tubules that allow production of CO_2_ in the pyrenoid (Mackinder *et al*., 2017; Burlacot *et al*., 2022; Mukherjee *et al*., 2019; Fig. 1). The production of protons by photosynthetic electron transport lowers the pH in the thylakoid tubules to produce CO_2_ (Raven, 1997; Burlacot & Peltier, 2023).

Pyrenoids are dynamic structures, transiently adjusting their physical properties in response to carbon availability. When CO_2_ is plentiful, the pyrenoid becomes smaller, maintaining a small core structure of tubules containing RuBisCO and pyrenoid structural proteins (Barrett *et al*., 2021). Pyrenoid assembly is facilitated by the phase‐separating protein ESSENTIAL PYRENOID COMPONENT 1 (EPYC1), which mediates RuBisCO condensation through physical interaction mediated by two alpha helices in the small subunit of RuBisCO (Atkinson *et al*., 2019).

These examples of discovering functions of metabolic biocondensates started with direct observation of the biocondensate in unlabeled cells. This approach cannot identify all metabolic biocondensates as it requires the biocondensate to be a large, electron‐dense cellular feature. More recently described metabolic biocondensates result from examining enzymes that are already biochemically characterized using cell biology techniques.

## III. Recently described metabolic biocondensates

Some enzymes in biochemically well‐studied pathways have been evaluated for their localization *in vivo* with a surprising result: several well‐known enzymes assemble into biocondensates. The biocondensates discussed below contain enzymes whose functions have been known for 25–100 years, including enzymes in purine biosynthesis (purinosome), glycolysis (glycolytic bodies or g‐bodies), and rhamnose biosynthesis (rhamnosome). Recent technological developments enabling direct visualization of enzyme localization through live imaging allowed researchers to classify these pathways as metabolic biocondensates (Table 1).

### Purinosome

**Fig. 2:**
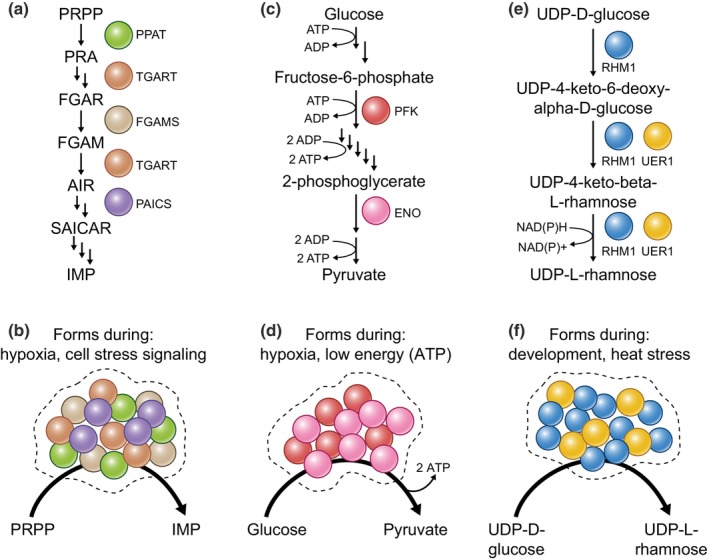
Function and regulation of recently found metabolic biocondensates. Illustrations representing the purinosome from mammalian systems (a, b), g‐bodies from yeast (*Saccharomyces cerevisiae*) and *Caenorhabditis elegans* (c, d), and rhamnosome from *Arabidopsis thaliana* (e, f) at the pathway (a, c, e) and cellular (b, d, f) levels. Pathway diagrams are simplified to only contain names for the starting and ending metabolites, as well as intermediates that are directly consumed or produced by enzymes reviewed here. Single reactions that produce intermediates are represented by a straight arrow. Abbreviations of metabolite names and enzymes: phosphoribosylpyrophosphate (PRPP), phosphoribosylpyrophosphate amidotransferase (PPAT), phosphoribosylamine (PRA), N‐formylglycinamide ribonucleotide (FGAR and FGAM), aminoimidazole ribonucleotide (AIR), N‐succinocarboxyamide‐5‐aminoimidazole ribonucleotide (SAICAR), inosine monophosphate (IMP), phosphofructokinase (PFK), enolase (ENO), RHAMNOSE BIOSYNTHESIS 1 (RHM1), uridine diphosphate‐4‐keto‐6‐deoxy‐D‐glucose‐3,5‐epimerase‐4‐reductase 1 (UER1), adenosine diphosphate (ADP), adenosine triphosphate (ATP), oxidized nicotinamide adenine dinucleotide phosphate (NADP+), and reduced nicotinamide adenine dinucleotide phosphate (NADPH).

Purinosomes are biocondensates critical for purine synthesis in eukaryotes. Although purine biosynthesis has been studied since the 1950s, researchers only validated the compartmentalization of purine biosynthesis in 2008 (An *et al*., 2008). Fluorescent tagging revealed that several purine biosynthetic enzymes formed cytoplasmic speckles, with strong interactions among the enzyme complex among bifunctional phosphoribosylaminoimidazole carboxylase and phosphoribosyl aminoimidazole succinocarboxamide synthase (PAICS), phosphoribosyl formylglycinamide synthase (FGAMS), amidophosphoribosyl transferase (PPAT), and trifunctional glycinamide ribonucleotide transformylase complex (TGART; Pedley *et al*., 2022; An *et al*., 2008; Fig. 2a). Mass spectrometry imaging showed metabolite channeling in purinosomes adjacent to mitochondria, providing evidence for their function as metabolic biocondensates whose activity can be regulated (Pareek *et al*., 2020). By clustering enzymes in the 10‐step pathway, purinosomes enhance substrate channeling, improving the efficiency of ATP and GTP synthesis, especially under stress or high‐energy demand (Doigneaux *et al*., 2020; Fig. 2b). Their assembly is dynamic and regulated by cellular cues, such as nutrient status and AMP‐activated protein kinase (AMPK) signaling (Schmitt *et al*., 2016). Once purine levels are restored, the purinosome disassembles. Purine biosynthesis is essential for life, and enzymes that synthesize purine are widely conserved, although purinosome formation and regulation remain unexplored in many organisms, including plants.

### Glycolytic bodies

G‐bodies are dynamic, membraneless structures that concentrate glycolytic enzymes to optimize ATP production, especially under stress (Jin *et al*., 2017). Although glycolysis has been studied for over a century, g‐bodies were only identified in 2017 as phase‐separated assemblies proposed to improve metabolic efficiency by minimizing diffusion and promoting substrate channeling (Jin *et al*., 2017). G‐bodies form in response to conditions that require fast ATP synthesis, such as hypoxia, nutrient scarcity, or high‐energy demand. G‐body assembly is mediated by protein–protein and protein‐RNA interactions that bring phosphofructokinase and enolase into proximity (Miura *et al*., 2013; Jin *et al*., 2017; Fuller *et al*., 2020; Fig. 2c,d). This organization allows cells to quickly modulate glycolysis as conditions change. G‐bodies are typically absent when energy is plentiful, with enzymes dispersed throughout the cytoplasm. However, in low‐oxygen or nutrient‐scarce environments, g‐bodies assemble rapidly (Miura *et al*., 2013; Jang *et al*., 2016; Jin *et al*., 2017; Fuller *et al*., 2020). Additionally, the enzymes within g‐bodies often form transient interactions that enable quick adjustment of glycolytic activity according to energy needs (Jang *et al*., 2016). G‐bodies may even alter their physical states, becoming more gel‐like or fluid‐like depending on cellular conditions to tune pathway activity (Fuller *et al*., 2020). As phosphofructokinase and enolase are broadly conserved enzymes, g‐body‐like structures might be conserved in diverse eukaryotes, but this has not been demonstrated to‐date. Whether the g‐body can functionally regulate metabolism in plants, for example, remains undemonstrated, and further study of g‐bodies holds promise to clarify how plants regulate energy production.

### Rhamnosome

Recent research examined the role of *Arabidopsis thaliana* RHAMNOSE BIOSYNTHESIS 1 (RHM1) condensation in uridine diphosphate (UDP)‐rhamnose synthesis (Field *et al*., 2024). UDP rhamnose is a building block of the cell wall and is involved in rhamnosylation of proteins and specialized metabolites (Mistou *et al*., 2016; Jiang *et al*., 2021). RHM1 enzymatic function and involvement in organismal development have been studied in plants for decades. RHM1 condensation was first described in 2009 when RHM1‐GFP was expressed in *Arabidopsis thaliana* and localized to small bodies in response to stress (Wang *et al*., 2009). RHM1 bodies were further identified in developing petals (Saffer *et al*., 2017) and were suggested to be localized to stress granules (Kosmacz *et al*., 2019). Recent work identified RHM1 bodies forming in leaves and the developing seed coat cells, which are strongly associated with an increased demand for UDP rhamnose (Field *et al*., 2024). Mutants in RHM1 that prevent body formation also prevent UDP‐rhamnose synthesis. Another component of the UDP‐rhamnose synthesis pathway, UER1, requires RHM1 body formation to function in UDP‐rhamnose synthesis, further suggesting that rhamnosome formation is strongly tied to UDP‐rhamnose synthesis (Field *et al*., 2024).

## IV. Emerging technologies for discovering metabolic biocondensates

To efficiently discover metabolic biocondensates, a cross‐disciplinary approach incorporating diverse technologies is needed (Table 2). Microscopic techniques, such as bimolecular fluorescence complementation (BiFC) and Förster (fluorescence) resonance energy transfer (FRET), are useful for demonstrating protein–protein interactions (Sekar & Periasamy, 2003; Kerppola, 2008). Cryo‐electron tomography (CryoET) imaging is a promising approach for determining the physical properties of biocondensates in cells (Doerr, 2017). For higher throughput protein–protein interaction‐based discovery, biochemical techniques, such as co‐fractionation mass spectrometry, cross‐linking mass spectrometry, and proximity labeling, have promise to demonstrate protein–protein interactions across diverse plant species (Zhu *et al*., 2016; Mair *et al*., 2019; McWhite *et al*., 2020; Liu *et al*., 2023, 2024; Luzarowski *et al*., 2023). Finally, fluorescently tagging enzymes at the genome level to produce enzyme localization atlases can accelerate the discovery of metabolic biocondensates (Wang *et al*., 2023).

**Table 2:** Promising new technologies for biocondensate discovery in plants.

Technology	Review of technology	Plant reference	Throughput	Key features
CryoET	Doerr (2017); Hong *et al*. (2023)	Otegui & Pennington (2019)	Low – Limited by sample preparation	Allows for high resolution imaging of unlabeled biocondensates
Proximity labeling	Qin *et al*. (2021)	Mair *et al*. (2019)	Low – Limited by requirement for compartment‐specific negative controls	Can detect weak or transient interactions
Co‐fractionation mass spectrometry	Luzarowski *et al*. (2023)	McWhite *et al*. (2020)	High	Labor‐intensive
Cross‐linking mass spectrometry	O'Reilly & Rappsilber (2018)	Zhu *et al*. (2016)	Medium – Limited by sample processing	Can be performed on untagged plants
BiFC	Kerppola (2008)	Schütze *et al*. (2009)	Low – Limited by time‐intensive imaging	Can only validate pairwise interactions
FRET	Sekar & Periasamy (2003)	Bücherl *et al*. (2014)	Low – Limited by time‐intensive imaging	Can only validate pairwise interactions
Yeast two‐hybrid	Brückner *et al*. (2009)	Ferro & Trabalzini (2013)	Medium/High – Limited by requirement to test bait and target proteins in pairs	Can only validate pairwise interactions
Genome‐scale enzyme tagging	Plant Cell Atlas Consortium *et al*. (2021)	Wang *et al*. (2023)	Medium/High – Limited by transformability of the plants or their cells	Labor‐intensive

BiFC, Bimolecular fluorescence complementation; FRET, Förster (fluorescence) resonance energy transfer.

## V. Considerations for engineering metabolic biocondensates

Recent efforts to engineer synthetic metabolic biocondensates in plants have been promising. A recent study improved pathway efficiency *in planta* through the formation of synthetic biocondensates (Lindstrom Battle *et al*., 2025). While localizing enzymes into metabolic biocondensates can improve pathway efficiency, it is not sufficient to optimize metabolic rates. Models exploring the relationship between biocondensate structure and efficiency suggest that an optimal ratio between enzymes in a compartmentalized pathway is required to maximize metabolic rates (Hinzpeter *et al*., 2017). Biocondensate size is another important determinant of efficiency, with a trade‐off existing between surface area and internal enzyme density (Hinzpeter *et al*., 2017). Finally, metabolic biocondensate efficiency can be limited when the substrate turnover rate exceeds the rate of substrate diffusion into the biocondensate (Hinzpeter *et al*., 2017). Efforts to engineer synthetic biocondensates should account for these limitations to meet the theoretical limit of pathway improvement via synthetic biocondensates.

## VI. Conclusion

Metabolic biocondensates play a direct role in regulating organismal physiology by enhancing metabolism in response to environmental and developmental cues. Applying cell biology approaches to studying metabolic enzymes and pathways is a promising avenue for discovering metabolic biocondensates and their roles in regulating metabolism (Plant Cell Atlas Consortium *et al.*, 2021). Plants remain a promising system for studying metabolic biocondensates with clear physiological impacts.

## Competing interests

None declared.

## Author contributions

EVS and SF contributed to the conceptualization, investigation, writing – original draft, writing – review and editing. EVS also contributed to visualization. SYR contributed to the conceptualization, funding acquisition, project administration, supervision, and writing – review and editing.

## Disclaimer

The New Phytologist Foundation remains neutral with regard to jurisdictional claims in maps and in any institutional affiliations.

## References

[nph70474-bib-0001] An S , Kumar R , Sheets ED , Benkovic SJ . 2008. Reversible compartmentalization of *de novo* purine biosynthetic complexes in living cells. Science 320: 103–106. 18388293 10.1126/science.1152241

[nph70474-bib-0002] Atkinson N , Velanis CN , Wunder T , Clarke DJ , Mueller‐Cajar O , McCormick AJ . 2019. The pyrenoidal linker protein EPYC1 phase separates with hybrid Arabidopsis‐Chlamydomonas Rubisco through interactions with the algal rubisco small subunit. Journal of Experimental Botany 70: 5271–5285. 31504763 10.1093/jxb/erz275 PMC6793452

[nph70474-bib-0003] Axen SD , Erbilgin O , Kerfeld CA . 2014. A taxonomy of bacterial microcompartment loci constructed by a novel scoring method. PLoS Computational Biology 10: e1003898. 25340524 10.1371/journal.pcbi.1003898 PMC4207490

[nph70474-bib-0004] Banani SF , Lee HO , Hyman AA , Rosen MK . 2017. Biomolecular condensates: organizers of cellular biochemistry. Nature Reviews. Molecular Cell Biology 18: 285–298. 28225081 10.1038/nrm.2017.7 PMC7434221

[nph70474-bib-0005] Barrett J , Girr P , Mackinder LCM . 2021. Pyrenoids: co‐fixing phase separated liquid organelles. Biochimica et Biophysica Acta, Molecular Cell Research 1868: 118949. 33421532 10.1016/j.bbamcr.2021.118949

[nph70474-bib-0006] Bassard JE , Halkier BA . 2018. How to prove the existence of metabolons? Phytochemistry Reviews 17: 211–227. 29755303 10.1007/s11101-017-9509-1 PMC5932110

[nph70474-bib-0007] Bobik TA , Havemann GD , Busch RJ , Williams DS , Aldrich HC . 1999. The propanediol utilization (pdu) operon of Salmonella Enterica Serovar Typhimurium LT2 includes genes necessary for formation of polyhedral organelles involved in coenzyme B12‐DEPENDENT 1,2‐propanediol degradation. Journal of Bacteriology 181: 5967–5975. 10498708 10.1128/jb.181.19.5967-5975.1999 PMC103623

[nph70474-bib-0008] Brückner A , Polge C , Lentze N , Auerbach D , Schlattner U . 2009. Yeast two‐hybrid, a powerful tool for systems biology. International Journal of Molecular Sciences 10: 2763–2788. 19582228 10.3390/ijms10062763 PMC2705515

[nph70474-bib-0009] Bücherl CA , Bader A , Westphal AH , Laptenok SP , Borst JW . 2014. FRET‐FLIM applications in plant systems. Protoplasma 251: 383–394. 24390247 10.1007/s00709-013-0595-7

[nph70474-bib-0010] Burlacot A , Dao O , Auroy P , Cuiné S , Li‐Beisson Y , Peltier G . 2022. Alternative photosynthesis pathways drive the algal CO_2_‐concentrating mechanism. Nature 605: 366–371. 35477755 10.1038/s41586-022-04662-9

[nph70474-bib-0011] Burlacot A , Peltier G . 2023. Energy crosstalk between photosynthesis and the algal CO_2_‐concentrating mechanisms. Trends in Plant Science 28: 795–807. 37087359 10.1016/j.tplants.2023.03.018

[nph70474-bib-0012] Cameron JC , Wilson SC , Bernstein SL , Kerfeld CA . 2013. Biogenesis of a bacterial organelle: the carboxysome assembly pathway. Cell 155: 1131–1140. 24267892 10.1016/j.cell.2013.10.044

[nph70474-bib-0013] Doerr A . 2017. Cryo‐electron tomography. Nature Methods 14: 34.

[nph70474-bib-0014] Doigneaux C , Pedley AM , Mistry IN , Papayova M , Benkovic SJ , Tavassoli A . 2020. Hypoxia drives the assembly of the multienzyme purinosome complex. The Journal of Biological Chemistry 295: 9551–9566. 32439803 10.1074/jbc.RA119.012175 PMC7363121

[nph70474-bib-0015] Ferro E , Trabalzini L . 2013. The yeast two‐hybrid and related methods as powerful tools to study plant cell signalling. Plant Molecular Biology 83: 287–301. 23794143 10.1007/s11103-013-0094-4

[nph70474-bib-0016] Field S , Dorone Y , Dwyer WP , Cox JA , Raba D , Froehlich J , Blea M *et al*. 2024. *Arabidopsis thaliana* RHAMNOSE 1 condensate formation drives UDP‐rhamnose synthesis. *bioRxiv*. doi: 10.1101/2024.02.15.580454.

[nph70474-bib-0017] Field S , Jang G‐J , Dean C , Strader LC , Rhee SY . 2023. Plants use molecular mechanisms mediated by biomolecular condensates to integrate environmental cues with development. Plant Cell 35: 3173–3186. 36879427 10.1093/plcell/koad062 PMC10473230

[nph70474-bib-0018] Fuller GG , Han T , Freeberg MA , Moresco JJ , Ghanbari Niaki A , Roach NP , Yates JR 3rd , Myong S , Kim JK . 2020. RNA promotes phase separation of glycolysis enzymes into yeast G bodies in hypoxia. eLife 9: e48480. 32298230 10.7554/eLife.48480 PMC7162659

[nph70474-bib-0019] He S , Crans VL , Jonikas MC . 2023. The pyrenoid: the eukaryotic CO_2_‐concentrating organelle. Plant Cell 35: 3236–3259. 37279536 10.1093/plcell/koad157 PMC10473226

[nph70474-bib-0020] Hinzpeter F , Gerland U , Tostevin F . 2017. Optimal compartmentalization strategies for metabolic microcompartments. Biophysical Journal 112: 767–779. 28256236 10.1016/j.bpj.2016.11.3194 PMC5340097

[nph70474-bib-0021] Holdsworth RH . 1971. The isolation and partial characterization of the pyrenoid protein of *Eremosphaera viridis* . The Journal of Cell Biology 51: 499–513. 5112653 10.1083/jcb.51.2.499 PMC2108136

[nph70474-bib-0022] Hong Y , Song Y , Zhang Z , Li S . 2023. Cryo‐electron tomography: the resolution revolution and a surge of *in situ* virological discoveries. Annual Review of Biophysics 52: 339–360. 10.1146/annurev-biophys-092022-100958 36719970

[nph70474-bib-0023] Jakobson CM , Tullman‐Ercek D , Slininger MF , Mangan NM . 2017. A systems‐level model reveals that 1,2‐propanediol utilization microcompartments enhance pathway flux through intermediate sequestration. PLoS Computational Biology 13: e1005525. 28475631 10.1371/journal.pcbi.1005525 PMC5438192

[nph70474-bib-0024] Jang S , Nelson JC , Bend EG , Rodríguez‐Laureano L , Tueros FG , Cartagenova L , Underwood K , Jorgensen EM , Colón‐Ramos DA . 2016. Glycolytic enzymes localize to synapses under energy stress to support synaptic function. Neuron 90: 278–291. 27068791 10.1016/j.neuron.2016.03.011 PMC4840048

[nph70474-bib-0025] Jeter RM . 1990. Cobalamin‐dependent 1,2‐propanediol utilization by *Salmonella typhimurium* . Journal of General Microbiology 136: 887–896. 2166132 10.1099/00221287-136-5-887

[nph70474-bib-0026] Jiang N , Dillon FM , Silva A , Gomez‐Cano L , Grotewold E . 2021. Rhamnose in plants – from biosynthesis to diverse functions. Plant Science 302: 110687. 33288005 10.1016/j.plantsci.2020.110687

[nph70474-bib-0027] Jin M , Fuller GG , Han T , Yao Y , Alessi AF , Freeberg MA , Roach NP , Moresco JJ , Karnovsky A , Baba M *et al*. 2017. Glycolytic enzymes coalesce in G bodies under hypoxic stress. Cell Reports 20: 895–908. 28746874 10.1016/j.celrep.2017.06.082 PMC5586494

[nph70474-bib-0028] Kerfeld CA , Aussignargues C , Zarzycki J , Cai F , Sutter M . 2018. Bacterial microcompartments. Nature Reviews. Microbiology 16: 277–290. 29503457 10.1038/nrmicro.2018.10 PMC6022854

[nph70474-bib-0029] Kerppola TK . 2008. Bimolecular fluorescence complementation (BiFC) analysis as a probe of protein interactions in living cells. Annual Review of Biophysics 37: 465–487. 10.1146/annurev.biophys.37.032807.125842 PMC2829326 18573091

[nph70474-bib-0030] Kosmacz M , Gorka M , Schmidt S , Luzarowski M , Moreno JC , Szlachetko J , Leniak E , Sokolowska EM , Sofroni K , Schnittger A *et al*. 2019. Protein and metabolite composition of arabidopsis stress granules. New Phytologist 222: 1420–1433. 30664249 10.1111/nph.15690

[nph70474-bib-0031] Laursen T , Borch J , Knudsen C , Bavishi K , Torta F , Martens HJ , Silvestro D , Hatzakis NS , Wenk MR , Dafforn TR *et al*. 2016. Characterization of a dynamic metabolon producing the defense compound Dhurrin in Sorghum. Science 354: 890–893. 27856908 10.1126/science.aag2347

[nph70474-bib-0032] Lindstrom Battle AL , Barrett AW , Fricker MD , Sweetlove LJ . 2025. Localising enzymes to biomolecular condensates increases their accumulation and benefits engineered metabolic pathway performance in *Nicotiana benthamiana* . *bioRxiv*. doi: 10.1101/2025.01.29.635479. PMC12854901 40203202

[nph70474-bib-0033] Liu C , Mentzelopoulou A , Hatzianestis IH , Tzagkarakis E , Skaltsogiannis V , Ma X , Michalopoulou VA , Romero‐Campero FJ , Romero‐Losada AB , Sarris PF *et al*. 2024. A proxitome‐RNA‐capture approach reveals that processing bodies repress coregulated hub genes. Plant Cell 36: 559–584. 37971938 10.1093/plcell/koad288 PMC10896293

[nph70474-bib-0034] Liu C , Mentzelopoulou A , Muhammad A , Volkov A , Weijers D , Gutierrez‐Beltran E , Moschou PN . 2023. An actin remodeling role for Arabidopsis processing bodies revealed by their proximity interactome. EMBO Journal 42: e111885. 36741000 10.15252/embj.2022111885 PMC10152145

[nph70474-bib-0035] Long BM , Förster B , Pulsford SB , Price GD , Badger MR . 2021. Rubisco proton production can drive the elevation of CO_2_ within condensates and carboxysomes. Proceedings of the National Academy of Sciences, USA 118: e2014406118. 10.1073/pnas.2014406118 PMC8106323 33931502

[nph70474-bib-0036] Luzarowski M , Sokolowska EM , Schlossarek D , Skirycz A . 2023. PROMIS: co‐fractionation mass spectrometry for analysis of protein‐metabolite interactions. Methods in Molecular Biology 2554: 141–153. 36178625 10.1007/978-1-0716-2624-5_10

[nph70474-bib-0037] Mackinder LCM , Chen C , Leib RD , Patena W , Blum SR , Rodman M , Ramundo S , Adams CM , Jonikas MC . 2017. A spatial interactome reveals the protein organization of the algal CO_2_‐concentrating mechanism. Cell 171: 133–147. 28938113 10.1016/j.cell.2017.08.044 PMC5616186

[nph70474-bib-0038] Mair A , Xu S‐L , Branon TC , Ting AY , Bergmann DC . 2019. Proximity labeling of protein complexes and cell‐type‐specific organellar proteomes in Arabidopsis enabled by TurboID. eLife 8: e47864. 31535972 10.7554/eLife.47864 PMC6791687

[nph70474-bib-0039] McWhite CD , Papoulas O , Drew K , Cox RM , June V , Dong OX , Kwon T , Wan C , Salmi ML , Roux SJ *et al*. 2020. A pan‐plant protein complex map reveals deep conservation and novel assemblies. Cell 181: 460–474. 32191846 10.1016/j.cell.2020.02.049 PMC7297045

[nph70474-bib-0040] Mistou M‐Y , Sutcliffe IC , van Sorge NM . 2016. Bacterial glycobiology: rhamnose‐containing cell wall polysaccharides in gram‐positive bacteria. FEMS Microbiology Reviews 40: 464–479. 26975195 10.1093/femsre/fuw006 PMC4931226

[nph70474-bib-0041] Miura N , Shinohara M , Tatsukami Y , Sato Y , Morisaka H , Kuroda K , Ueda M . 2013. Spatial reorganization of *Saccharomyces cerevisiae* enolase to alter carbon metabolism under hypoxia. Eukaryotic Cell 12: 1106–1119. 23748432 10.1128/EC.00093-13 PMC3754543

[nph70474-bib-0042] Mucha S , Heinzlmeir S , Kriechbaumer V , Strickland B , Kirchhelle C , Choudhary M , Kowalski N , Eichmann R , Hückelhoven R , Grill E *et al*. 2019. The formation of a camalexin biosynthetic metabolon. Plant Cell 31: 2697–2710. 31511315 10.1105/tpc.19.00403 PMC6881122

[nph70474-bib-0043] Mukherjee A , Lau CS , Walker CE , Rai AK , Prejean CI , Yates G , Emrich‐Mills T , Lemoine SG , Vinyard DJ , Mackinder LCM *et al*. 2019. Thylakoid localized bestrophin‐like proteins are essential for the CO_2_ concentrating mechanism of *Chlamydomonas reinhardtii* . Proceedings of the National Academy of Sciences, USA 116: 16915–16920. 10.1073/pnas.1909706116 PMC6708349 31391312

[nph70474-bib-0044] Nakayama T , Takahashi S , Waki T . 2019. Formation of flavonoid metabolons: functional significance of protein–protein interactions and impact on flavonoid chemodiversity. Frontiers in Plant Science 10: 821. 31338097 10.3389/fpls.2019.00821 PMC6629762

[nph70474-bib-0045] O'Reilly FJ , Rappsilber J . 2018. Cross‐linking mass spectrometry: methods and applications in structural, molecular and systems biology. Nature Structural & Molecular Biology 25: 1000–1008. 10.1038/s41594-018-0147-0 30374081

[nph70474-bib-0046] Otegui MS , Pennington JG . 2019. Electron tomography in plant cell biology. Microscopy 68: 69–79. 30452668 10.1093/jmicro/dfy133

[nph70474-bib-0047] Pareek V , Tian H , Winograd N , Benkovic SJ . 2020. Metabolomics and mass spectrometry imaging reveal channeled *de novo* purine synthesis in cells. Science 368: 283–290. 32299949 10.1126/science.aaz6465 PMC7494208

[nph70474-bib-0048] Pedley AM , Pareek V , Benkovic SJ . 2022. The Purinosome: a case study for a mammalian metabolon. Annual Review of Biochemistry 91: 89–106. 10.1146/annurev-biochem-032620-105728 PMC9531488 35320684

[nph70474-bib-0049] Plant Cell Atlas Consortium , Jha SG , Borowsky AT , Cole BJ , Fahlgren N , Farmer A , Huang SC , Karia P , Libault M , Provart NJ *et al*. 2021. Vision, challenges and opportunities for a Plant Cell Atlas. eLife 10: e66877. 34491200 10.7554/eLife.66877 PMC8423441

[nph70474-bib-0050] Price GD , Badger MR . 1991. Evidence for the role of carboxysomes in the cyanobacterial CO_2_‐concentrating mechanism. Canadian Journal of Botany 69: 963–973.

[nph70474-bib-0051] Qin W , Cho KF , Cavanagh PE , Ting AY . 2021. Deciphering molecular interactions by proximity labeling. Nature Methods 18: 133–143. 33432242 10.1038/s41592-020-01010-5 PMC10548357

[nph70474-bib-0052] Rae BD , Long BM , Badger MR , Price GD . 2013. Functions, compositions, and evolution of the two types of carboxysomes: polyhedral microcompartments that facilitate CO_2_ fixation in cyanobacteria and some proteobacteria. Microbiology and Molecular Biology Reviews 77: 357–379. 24006469 10.1128/MMBR.00061-12 PMC3811607

[nph70474-bib-0053] Raven JA . 1997. CO_2_‐concentrating mechanisms: a direct role for thylakoid lumen acidification? Plant, Cell & Environment 20: 147–154.

[nph70474-bib-0054] Saffer AM , Carpita NC , Irish VF . 2017. Rhamnose‐containing cell wall polymers suppress helical plant growth independently of microtubule orientation. Current Biology 27: 2248–2259. 28736166 10.1016/j.cub.2017.06.032

[nph70474-bib-0055] Sampson EM , Bobik TA . 2008. Microcompartments for B12‐dependent 1,2‐propanediol degradation provide protection from DNA and cellular damage by a reactive metabolic intermediate. Journal of Bacteriology 190: 2966–2971. 18296526 10.1128/JB.01925-07 PMC2293232

[nph70474-bib-0056] Schmitt DL , Cheng Y‐J , Park J , An S . 2016. Sequestration‐mediated downregulation of *de novo* purine biosynthesis by AMPK. ACS Chemical Biology 11: 1917–1924. 27128383 10.1021/acschembio.6b00039 PMC5675104

[nph70474-bib-0057] Schütze K , Harter K , Chaban C . 2009. Bimolecular fluorescence complementation (BiFC) to study protein–protein interactions in living plant cells. Methods in Molecular Biology 479: 189–202. 19083187 10.1007/978-1-59745-289-2_12

[nph70474-bib-0058] Sekar RB , Periasamy A . 2003. Fluorescence resonance energy transfer (FRET) microscopy imaging of live cell protein localizations. The Journal of Cell Biology 160: 629–633. 12615908 10.1083/jcb.200210140 PMC2173363

[nph70474-bib-0059] Shively JM , Ball F , Brown DH , Saunders RE . 1973. Functional organelles in prokaryotes: polyhedral inclusions (carboxysomes) of *Thiobacillus neapolitanus* . Science 182: 584–586. 4355679 10.1126/science.182.4112.584

[nph70474-bib-0060] Shively JM , Bradburne CE , Aldrich HC , Bobik TA , Mehlman JL , Jin S , Baker SH . 1998. Sequence homologs of the carboxysomal polypeptide CsoS1 of the thiobacilli are present in Cyanobacteria and enteric bacteria that form carboxysomes – polyhedral bodies. Canadian Journal of Botany 76: 906–916.

[nph70474-bib-0061] Shively JM , Decker GL , Greenawalt JW . 1970. Comparative ultrastructure of the Thiobacilli. Journal of Bacteriology 101: 618–627. 5413830 10.1128/jb.101.2.618-627.1970 PMC284949

[nph70474-bib-0062] Smith GK , Mueller WT , Wasserman GF , Taylor WD , Benkovic SJ . 1980. Characterization of the enzyme complex involving the folate‐requiring enzymes of *de novo* purine biosynthesis. Biochemistry 19: 4313–4321. 7417406 10.1021/bi00559a026

[nph70474-bib-0063] Sutter M , Melnicki MR , Schulz F , Woyke T , Kerfeld CA . 2021. A catalog of the diversity and ubiquity of bacterial microcompartments. Nature Communications 12: 3809. 10.1038/s41467-021-24126-4 PMC8217296 34155212

[nph70474-bib-0064] Toraya T , Honda S , Kuno S , Fukui S . 1978. Coenzyme B12‐dependent diol dehydratase: regulation of apoenzyme synthesis in *Klebsiella pneumoniae* (Aerobacter Aerogenes) ATCC 8724. Journal of Bacteriology 135: 726–729. 210157 10.1128/jb.135.2.726-729.1978 PMC222436

[nph70474-bib-0065] Toyokawa C , Yamano T , Fukuzawa H . 2020. Pyrenoid starch sheath is required for LCIB localization and the CO_2_‐concentrating mechanism in green algae. Plant Physiology 182: 1883–1893. 32041908 10.1104/pp.19.01587 PMC7140920

[nph70474-bib-0066] Vaucher JP . 1803. Histoire Des Conferves D'eau Douce. A Genève, Switzerland: Chez J. J. Paschoud, Libraire.

[nph70474-bib-0067] Wang J , Ji Q , Jiang L , Shen S , Fan Y , Zhang C . 2009. Overexpression of a cytosol‐localized Rhamnose biosynthesis protein encoded by Arabidopsis RHM1 gene increases Rhamnose content in cell wall. Plant Physiology and Biochemistry 47: 86–93. 19056285 10.1016/j.plaphy.2008.10.011

[nph70474-bib-0068] Wang L , Patena W , Van Baalen KA , Xie Y , Singer ER , Gavrilenko S , Warren‐Williams M , Han L , Harrigan HR , Hartz LD *et al*. 2023. A chloroplast protein atlas reveals punctate structures and spatial organization of biosynthetic pathways. Cell 186: 3499–3518. 37437571 10.1016/j.cell.2023.06.008

[nph70474-bib-0069] Wang Y , Stessman DJ , Spalding MH . 2015. The CO_2_ concentrating mechanism and photosynthetic carbon assimilation in limiting CO_2_: how Chlamydomonas works against the gradient. The Plant Journal: For Cell and Molecular Biology 82: 429–448. 25765072 10.1111/tpj.12829

[nph70474-bib-0070] Zhang Y , Fernie AR . 2020. Metabolons, enzyme‐enzyme assemblies that mediate substrate channeling, and their roles in plant metabolism. Plant Communications 2: 100081. 33511342 10.1016/j.xplc.2020.100081 PMC7816073

[nph70474-bib-0071] Zhu X , Yu F , Yang Z , Liu S , Dai C , Lu X , Liu C , Yu W , Li N . 2016. *In planta* chemical cross‐linking and mass spectrometry analysis of protein structure and interaction in Arabidopsis. Proteomics 16: 1915–1927. 27198063 10.1002/pmic.201500310

